# Anastomotic leakage following resection of the esophagus—introduction of an endoscopic grading system

**DOI:** 10.1186/s12957-022-02551-z

**Published:** 2022-03-31

**Authors:** Jeannine Bachmann, Marcus Feith, Christoph Schlag, Mohamed Abdelhafez, Marc E. Martignoni, Helmut Friess

**Affiliations:** 1 Department of Surgery, Klinikum rechts der Isar, Technische Universität München, 81675 Munich, Germany; 2 Department of Internal Medicine, Klinikum rechts der Isar, Technische Universität München, Ismaningerstr, 22, 81675 Munich, Germany

**Keywords:** Anastomotic leakage, Endoscopic grading system, Endoluminal vacuum therapy

## Abstract

**Background:**

Malignant tumors of the esophagus are the sixth leading cause of cancer-related deaths worldwide. Postoperative leakage of the esophago-gastrostomy leads to mediastinal sepsis, which is still associated with a high morbidity and mortality rate. The aim of this study was to describe the endoscopic view of the different severity grades of an anastomotic leakage.

**Methods:**

Patients

Between June 2016 and September 2018, 144 patients were operated upon in the Department of Surgery, University of Munich, Germany.

Among these patients, 34 (23.6%) presented with a leakage of the anastomosis.

Endoscopy

In this retrospective analysis, the focus is to describe different patterns of leakage of the anastomosis.

**Results:**

We studied 34 patients in whom post-esophagectomy leakage of the anastomosis was detected and treated with an endoluminal vacuum sponge system. The leakage healed in 26 of 29 patients (success rate 89.7%).

With the increasing severity of leakage, the treatment time and the in-hospital mortality correspondingly increased. Furthermore, the incidence of the development of a fistula to the tracheobronchial system increased with higher grades of leakage.

**Conclusions:**

Exact descriptions of leakage are necessary to compare the cases and to prove post-treatment improvement. This is, to our knowledge, the first publication to present a leakage grading score in patients after esophagectomy including reconstruction with a gastric tube.

This new grading system needs to be tested in further analyses, with a special focus on prospective analysis.

## Introduction

Malignant tumors of the esophagus are the sixth leading cause of cancer-related deaths worldwide [1, 2]. The operative procedure in patients with cancer of the esophagus and the esophagogastric junction—a right abdominothoracic esophagectomy with intrathoracic anastomosis and two-field lymphadenectomy—is still demanding due to a high morbidity and mortality rate. Postoperative leakage of the esophago-gastrostomy leads to mediastinal sepsis, which is still associated with a high morbidity and mortality rate; morbidity rates of up to 73% are reported [3, 4]. Furthermore, a hospital mortality of up to 9.6% is reported [3, 5]. A recent meta-analysis, which included the data of 11,368 patients with esophageal cancer, revealed a significant correlation of postoperative anastomotic leakage with a decreased 5-year survival as well as with a decreased 5-year disease-free survival [6]. One major factor contributing to morbidity as well as mortality is leakage of the anastomosis, which is reported in up to 23% of cases [3, 5, 7, 8]. In addition, anastomotic leakage after resection of the esophagus or the esophagogastric junction has a significant negative effect on long-term survival [5, 6].

However, what exactly can be termed a leakage of the esophagogastric anastomosis? There is still no clear definition; especially, no grading is available. To compare different patient collectives and to define the best treatment standards for different degrees of anastomotic leakage, a pragmatic and clear definition is needed.

Furthermore, the best method to detect leakage is still under discussion [9]. Additionally, the appropriate treatment of an anastomotic leakage following esophagectomy is still being debated. Vacuum-assisted closure is a method first described in 2008 by Wedemeyer et al. [10]. Several reports have investigated the treatment of anastomotic leakages using this system; success rates up to 88% are reported [8, 11, 12].

The aim of this study was to define a leakage of the esophago-gastric anastomosis by using endoscopy and to describe the endoscopic view of the different severity grades of an anastomotic leakage.

## Patients and methods

### Patients

Between June 2016 and September 2018, 144 patients were operated upon in the Department of Surgery, University of Munich, Germany. Among these patients, 34 (23.6%) presented with a leakage of the anastomosis; a vacuum-assisted closure system was used to treat the leakage in these patients. Eight patients had squamous cell cancer of the esophagus, 25 had adenocarcinoma of the distal esophagus, and one had achalasia. In 26 patients (76.5%), neoadjuvant treatment was used (14 patients received only chemotherapy before the operation, while 12 received combined radio-chemotherapy). Every patient gave written informed consent to publish data in an anonymous matter. All data evaluated in this study were acquired in clinical routine. The study was performed according to the guidelines of the Declaration of Helsinki. The ethics committee of the faculty of medicine of the technical university, Munich, gave admission to informed consent and data acquisition (553/15 S)

### Operative procedure

The first operative step is the incision of the upper epigastrium for mobilization of the stomach, followed by the formation of a gastric tube along the greater curvature. Lymphadenectomy along the celiac axis and parapancreatic region is performed. Then, abdominotransthoracic en bloc esophagectomy is done through a right transthoracic approach, including lymphadenectomy in the upper mediastinum. The reconstruction of the intestinal passage is finished with an esophagogastrostomy using the circular stapling method [13]. After esophagectomy, endoscopy was performed in cases where signs indicating possible leakages, such as fever or a change in the drainage fluid, were noted.

### Endoscopy

In this study, the endoscopic view in the postoperative course after esophagectomy and reconstruction with gastric tube is presented. The focus is to describe different patterns of leakage of the anastomosis. The analysis is retrospective.

**Fig. 1:**
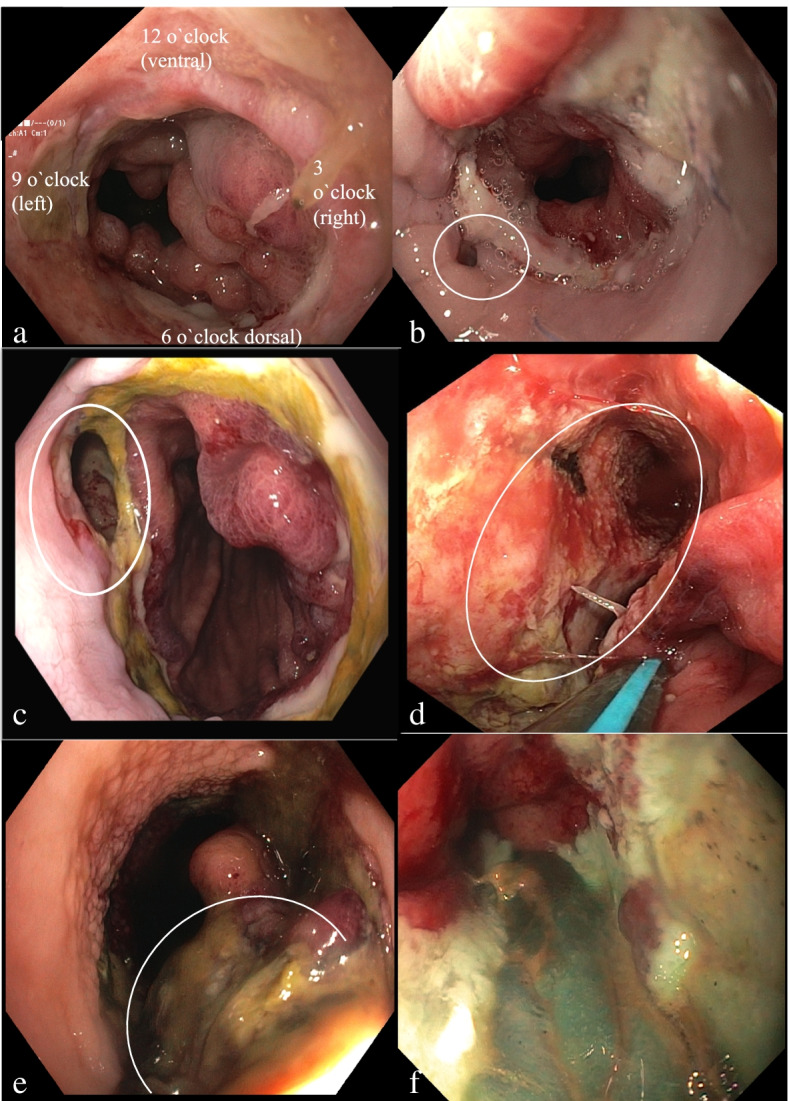
Endoscopic views with regard to leakage grading. **a** Anastomosis on day 6 after the operation. **b** Leakage grade 1a: dehiscence of the anastomosis less than a quarter of the circumference without a cavity. **c** Leakage grade 1b: rosy tubular stomach, dehiscence of the anastomosis less than a quarter of the circumference, with a cavity. **d** Leakage grade 2: rosy tubular stomach, dehiscence of the anastomosis more than a quarter of the circumference, with a cavity. **e** Leakage grade 3: the necrotic mucosal layer of the tubular stomach. **f** Leakage grade 4: the necrotic wall of the tubular stomach

First, the anastomosis is described clockwise: the 12 o’clock position on the endoscopic view is the localized ventral (retrosternal) aspect of the anastomosis; at the 3 o’clock position, the inverted blind stump is located; and at the 6 o’clock position, the dorsal part of the anastomosis is located. Table 1 provides an overview of the examined data. Figure 1a gives an endoscopic view of an anastomosis on day 5 after the operation.

**Table 1:** Endoscopic view of the anastomosis and the tubular stomach

	Appearance
Leakage	12–3 o’clock quadrant
	3–6 o’clock quadrant
	6–9 o’clock quadrant
	9–12 o’clock quadrant
Tubular stomach	Rosy
	Necrotic mucosa
	Necrotic wall
Dehiscence	Without cavity
	With cavity
Anastomosis	Rosy
	Partial necrotic < ¼ circumference
	Necrotic > ¼ circumference
	Necrotic whole circumference

If a dehiscence of the anastomosis is detected, its location and size are both documented. If a cavity is present, its presence is noted and its dimensions, including depth, are mentioned. The mucosa and the gastric tube wall are checked for signs of perfusion or necrosis. If there is necrotic and/or fibrotic tissue in the anastomotic region and dehiscence of less than a quadrant of the circumference is detected, a grade 1 leakage is diagnosed. This grade is furthermore separated into grade 1 a and b, respectively. Grade 1a is diagnosed if the anastomosis is present without a cavity (Fig. 1b). In case of a leakage less than a quarter of the circumference with a cavity, grade 1b is diagnosed (Fig. 1c shows the endoscopic finding).

Grade 2 is diagnosed if dehiscence of the anastomosis exceeds a quarter of the circumference, in the absence of a necrotic mucosa or necrotic wall of the tubular stomach (see Fig. 1d). If necrosis in the mucosa of the gastric tube is seen, grade 3 is diagnosed (Fig. 1e illustrates this endoscopic finding), whereas grade 4 is stated if necrosis of the whole tubular wall of the stomach is detected (Fig. 1f). Using this exact description and localization of the dehiscence, it is possible to compare the results of the following endoscopic examinations. Table 2 shows an overview of the descriptions of the endoscopic findings, Fig. 1a–f presents the different endoscopic findings.

**Table 2:** Grade of leakage and endoscopic appearance

Grade	Dehiscence of anastomosis	Cavity	Tubular stomach
1a	< ¼ of the circumference	No	Rosy
1b	< ¼ of the circumference	Yes	Rosy
2	> ¼ of the circumference	Yes	Rosy
3	Yes/no	Yes/no	Necrotic mucosa
4	Yes/no	Yes/no	Necrotic wall

After the first detection of a leakage of the anastomosis, a sponge is placed endoscopically. This sponge is placed in the lumen of the tubular stomach/esophagus, if there is no cavity or a dehiscence of the anastomosis less than a quarter of the circumference detected whereas on the other hand, it is placed in the cavity, if the leakage of the anastomosis exceeds a quarter of the circumference a quarter of the circumference with the underlying cavity, respectively: the different grade implies different placement of the endoscopically introduced sponge. The system is taken out routinely every 2 to 3 days to have the possibility to check the healing process and to renew the sponge. If there is a good process to detect, the sponge can be removed. If there is no or only little granulation tissue present over several days, **and** in the absence of necrotic tissue, the change of the procedure is to be considered. In these situations, the decision has to be to take out the sponge and to describe the leakage again: when there is a punctual leakage, an over the scope clip is inserted; if there is a broader leakage, the insertion of a self-expanding fully covered stent was performed.

**Fig. 2:**
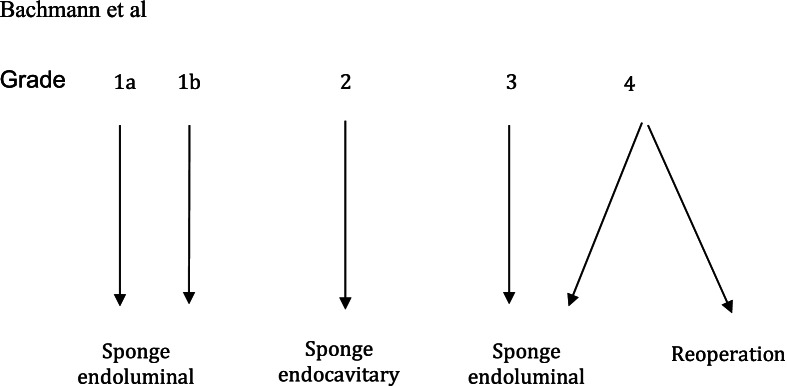
Algorithm: detection of leakage severity and recommended treatment

In our analysis, the different grades lead to different treatment options. In patients with leakage of grade 1a and 1b, the sponge was placed into the lumen of the esophagus and tubular stomach, respectively. In patients with grade 2 leakage, the vacuum system was placed into the cavity after wound debridement. In patients with grade 3 leakage, the sponge was placed into the lumen of the tubular stomach. In patients with grade 4, reoperation is mostly considered; if this is not possible due to the patient’s condition, an endoluminal vacuum treatment is applied. Figure 2 presents an algorithm that is used to determine where the sponge should be placed: in the lumen of the gastric tube or the cavity.

Table 2 provides a grading system that takes all the abovementioned parameters into account to show the severity of the leakage.

### Statistical analysis

Statistical analysis was performed using SPSS software, version 25 (SPSS Inc., Chicago, IL, USA). Results are displayed in median [with lower and upper quartile]. For testing significant differences between the examined groups, we used Student’s *t* test and the Mann-Whitney *U* test. A significance level < 0.05 was used.

## Results

Between June 2016 and August 2018, in the Department of Surgery, University of Munich, 34 patients presented with leakage of the anastomosis after esophagectomy with reconstruction with a gastric tube, in whom a vacuum-assisted closure system was used. No fistulae were detected on the initial endoscopic examination after the operation.

The median day of detection of the leakage was day 6 after the operation (5/9 lower/upper quartile). During the postoperative course, 5 (14.7%) patients died. The data of these patients were excluded from further analysis.

To present the different clinical courses of the patients depending on the scoring system, the data are presented for every severity grade in detail.

### Grade 1a

Twelve patients presented with leakage of grade 1a severity. In these, the sponge was placed into the esophageal lumen and tubular stomach and was changed after 2 to 3 days. The in-hospital mortality was 8.3%. The median number of days of treatment with the vacuum system was 15 (9/31 lower/upper quartile). The clinical success rate was 100% (11/11). In 8 patients (72.7%), only the sponge treatment was needed with regard to the leakage. In 2 patients (18.2%), after extracting the sponge, a clip was applied. In one patient, a self-expanding stent was used after the sponge treatment; the stent was removed 4 weeks later.

In all cases, an endoscopic check as well as a contrast swallow radiograph was performed to determine treatment success.

### Grade 1b

Ten patients presented with leakage of grade 1b severity. In these patients, the sponge was placed into the esophageal lumen and tubular stomach and was changed after 2 to 3 days. The in-hospital mortality reached 10% in grade 1 b (1/10).

The median number of days of treatment was 25 (14/35 lower/upper quartile). The success rate in grade 1b was 88.9% (8/9). One patient developed a fistula to the tracheobronchial system. In 4 patients (44.4%), the leakage healed with the endoluminal sponge treatment; in 2 patients, a self-expanding stent was applied (22.2%); and in 2 patients, a clip was used to close the leakage after treatment with the endoluminal vacuum system (22.2%).

### Grade 2

In 8 patients with a dehiscence of the anastomosis > ¼ circumference with an existing cavity, the sponge was placed into the cavity after the cavity was debrided. One patient (12.5%) died within thirty postoperative days.

The median treatment period was 27 days (18/34 lower/upper quartile). The success rate in grade 2 was 71.4% (5/7); in 4 patients (57.1%), the leakage healed with the endoluminal sponge treatment, while in 1 patient, a self-expanding stent was used after the extraction of the sponge. Two patients developed a fistula to the tracheobronchial system during the postoperative course.

### Grade 3

Two patients presented with grade 3 leakage. The vacuum-assisted closure system was placed into the lumen in both patients. During the postoperative course, one of these patients died.

### Grade 4

Two patients were classified as having grade 4 leakage; both were treated with an endoluminal placed vacuum-assisted closure system. One patient died within 30 days postoperatively.

## Discussion

Anastomotic leakage after esophagectomy with reconstruction with a tubular stomach remains a severe complication with a high risk for mortality and morbidity. This is, to our knowledge, the first publication in which the leakage is described with different severity grades as determined based on the endoscopic view.

We studied 34 patients in whom post-esophagectomy leakage of the anastomosis was detected and treated with an endoluminal vacuum sponge system. The overall mortality rate was 14.7% (5/34); the data of these patients were excluded from further analyses. In Tables 3 and 4, the data are presented with regard to the severity.

**Table 3:** Mortality and development of a fistula to the tracheobronchial with regard to grading

Grade	Patients, *N*	Periop. mortality, % (*N*)	Development of fistulae, *N*
1a	12	8.3 (1)	0
1b	10	10 (1)	1
2	8	12.5 (1)	2
3	2	n.d. (*N* = 1)	1
4	2	n.d. (*N* = 1)	0

**Table 4:** Severity of leakage with duration of treatment

Grade	Patients, *N*	Median duration of treatment (days)	Healing of leakage, % (*n*)
1a	11	15 (9/31)	100 (11)
1b	9	25 (14/35)	88.9 (8)
2	7	27 (18/34)	71.4 (5)
3	1	n.d. (*N* = 1)	n.d. (*N* = 1)
4	1	n.d. (*N* = 1)	n.d. (*N* = 1)

Median treatment duration including lower/upper quartile and success rate (*N* = 29) is demonstrated. Patients who died during the postoperative course are excluded

*n.d.* not done

The leakage healed in 26 of 29 patients (success rate 89.7%). In 16 (61.5%) patients, the sponge system alone was successful, while in 6 (23.1%), a self-expanding stent was used after treatment with the sponge. In the remaining 4 (15.4%) patients, a clip was applied. A contrast swallow proved the success of the treatment in all patients. Endoscopic suturing devices are widely used in bariatric surgery and to close large mucosal defects after endoscopic submucosal resections. The use in postoperative leakages, in which the extent of the defect is in some patients, is getting greater despite the endoscopic treatment over time and with the experience of necrotic margins of the tissue, in which there is no good situation to use an endoscopic suturing system, we do not use this device in these patients.

Due to a small number of patients in the grade 3 and 4 categories, we compared the following data only in groups 1a, 1b, and 2. With the increasing severity of leakage, the treatment time and the in-hospital mortality correspondingly increased. As the leakage grade increased, the healing success rate decreased: in grade 1a cases, the success rate was 100%, which reduced to 88.9% in grade 1b and 71.4% in grade 2.

## Conclusion

Exact descriptions of leakage are necessary to compare the cases and to prove post-treatment improvement. This is, to our knowledge, the first publication to present a leakage grading score in patients after esophagectomy including reconstruction with a gastric tube.

This new grading system needs to be tested in further analyses, with a special focus on prospective analysis.

## Data Availability

The underlying data of this retrospective analysis are saved in an anonymous matter in a data bank.
